# Incidental Finding and Endovascular Repair of a Left Internal Mammary Artery Aneurysm following a Multivessel Coronary Artery Bypass Graft

**DOI:** 10.1155/2021/8831235

**Published:** 2021-02-17

**Authors:** Osayi Lawani, Jiries Ganim, Rick Ganim

**Affiliations:** ^1^HCA Houston Healthcare-Kingwood, USA; ^2^University of Southern California, USA; ^3^Vital Heart & Vein, USA

## Abstract

True aneurysms discovered within the internal mammary artery are extremely rare and typically have an asymptomatic occurrence. Their presentation and management have also been variable due to their low incidence, decreased detection, or lack of documentation. They have a high risk for morbidity or mortality as they can possibly rupture with increasing size and thus become life-threatening. Coronary CT angiography is the most definitive test for confirming and finding complications related to the aneurysm. With an increase in the aging population and advancement in the techniques used in coronary artery bypass grafting, it is likely that the rate of recorded occurrence of aneurysms and pseudoaneurysms will increase. Endovascular repair is currently the most favored treatment modality. In this report, we describe a case of a 74-year-old male who was incidentally found to have a left internal mammary artery aneurysm following complaints of chest pain related to another nearly occluded grafted vessel. To the best of our knowledge, and following an extensive literature review, this is likely the first documented case of a true aneurysm found within a left internal mammary artery bypass graft. The patient recovered well following placement of a covered stent; however, upon follow-up one year later, he was found to have stenosis of the same vessel, which was subsequently treated without further complication.

## 1. Introduction

Aneurysms manifesting from the internal mammary artery (IMA) are a very rare occurrence. Although they are usually small, these aneurysms will have an increased risk of rupture which can be life-threatening [1]. Other possible complications include hemothorax, thromboembolism, compression of adjacent structures, myocardial infarction, and possible death. Aneurysms in the IMA are typically found incidentally on cardiac angiography. Characteristically, these aneurysms grow slowly and silently, making this a highly underdiagnosed disease process [2].

The left IMA (LIMA) is the vessel used most to bypass a significant stenosis of the left anterior descending (LAD) artery [3]. Advantages of using the LIMA over a saphenous vein graph are long-term patency and survival rates greater than 90% beyond 10 years and decreased postoperative mortality [4]. The exact cause of an IMA aneurysm is unknown, however, it can be traumatic or iatrogenic in origin, caused by progressive atherosclerosis, or due to connective tissue disorders such as Marfan, Loeys-Dietz, and Ehlers-Danlos syndromes [5]. In this report, we present a case of an elderly male who was found to have a large aneurysm located in a LIMA to LAD artery graft following a coronary bypass procedure 6 months prior.

## 2. Case History

A 74-year-old male with a past medical history of coronary artery disease with cardiac stenting, peripheral artery disease with femoral popliteal bypass, hypertension, hyperlipidemia, diabetes mellitus, chronic kidney disease, deep vein thrombosis, and distant history of heavy tobacco use, presented to a local emergency department with complaints of symptoms that were suggestive of unstable angina. Cardiology was consulted and a left heart catheterization was performed.

The left main had mild diffuse plaquing. The LAD was of large caliber, and in the midsection at the takeoff of a large diagonal branch, there were stents in the artery and in the ostium of the diagonal branch, which had about 60% and 90% in-stent restenosis, respectively. The left circumflex artery was small in caliber, and in the proximal section there was 85% stenosis. The ostium of the first obtuse marginal branch had 70% stenosis. The right coronary artery was occluded in its midsegment, and the distal coronary system was able to perfuse left-to-right collateral circulation. Finally, the left internal mammary artery was widely patent. Given the multivessel coronary artery disease and small caliber of the left circumflex artery, it was recommended that the patient be evaluated by cardiothoracic surgery for a coronary artery bypass graft (CABG).

Six months later, the patient presented to his cardiologist with complaints of intermittent episodes of nitroglycerin-responsive, substernal chest pain that radiated down both arms with strenuous activity. The patient had undergone a six-vessel CABG five months earlier without any residual complications to date. Home medications included dual antiplatelet therapy, antihypertensives, antianginal agents, and a lipid-lowering agent. An electrocardiogram showed normal sinus rhythm, occasional premature atrial contractions, and no ischemic changes. Laboratory results showed elevated troponin I. He was subsequently admitted to the hosptial for cardiac catheterization.

**Figure 1:**
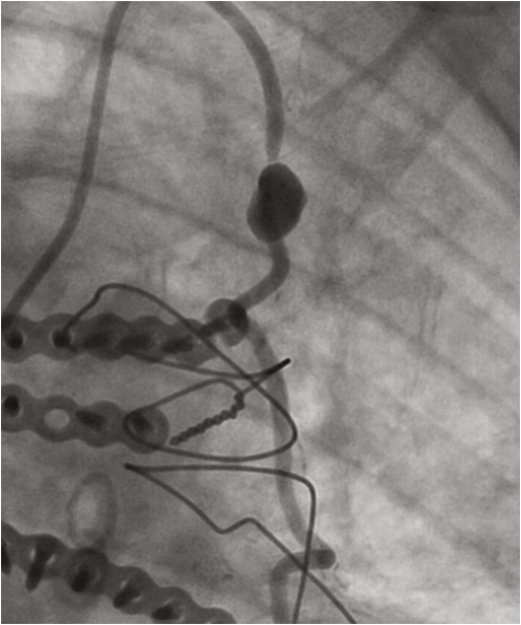
Large aneurysm in the left internal mammary artery to the left anterior descending artery bypass graft.

The distal left main had 40-50% stenosis just proximal to the bifurcation of the LAD and left circumflex arteries. The proximal-to-mid portion of the LAD continued to have 60% in-stent restenosis. The ostium of the diagonal branch also continued to have 90% in-stent restenosis. The proximal portion of the left circumflex had increased stenosis from 85% to 99%, and the midsegment was now occluded. The right coronary artery remained occluded in its midsegment with collateral circulation. A saphenous vein graft to the right coronary artery had 50% stenosis. A saphenous vein graft to the obtuse marginal branch in the mid-to-distal segment had 95% stenosis. A LIMA graph to the LAD was patent; however, there was a large aneurysm within the LIMA with TIMI-3 blood flow (Figure 1). The culprit vessel causing the intermittent episodes of chest pain was felt to be the saphenous vein graph to the obtuse marginal branch, and a drug-eluting stent was placed.

**Figure 2:**
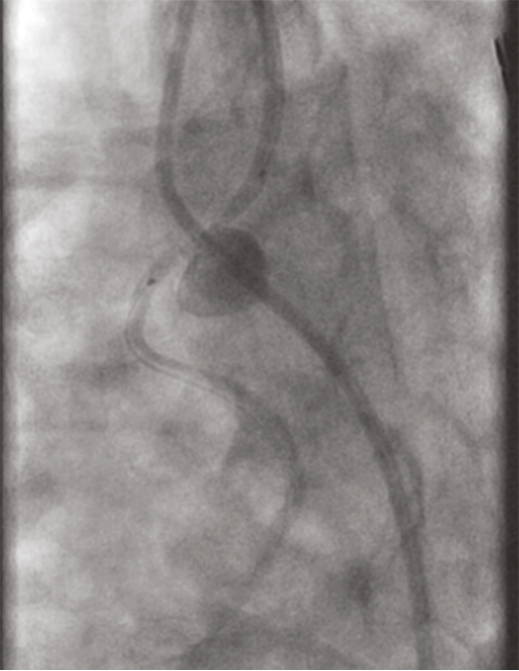
Covered stent placement in the midsegment of a large left internal mammary artery bypass graft aneurysm.

**Figure 3:**
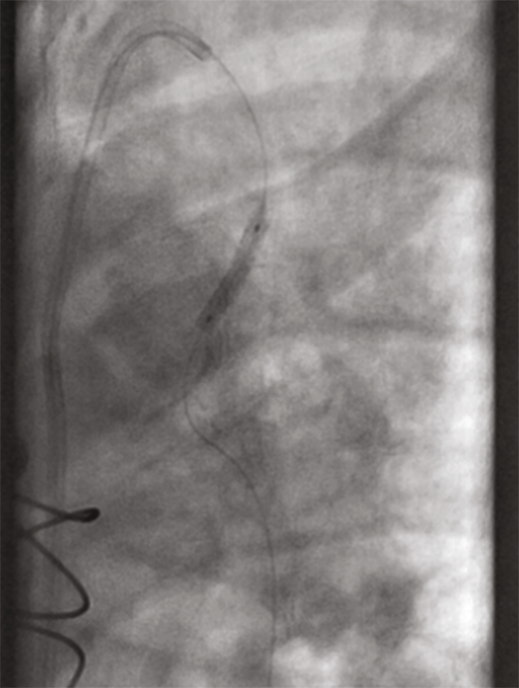
Stent placement within the left internal mammary artery bypass graft.

**Figure 4:**
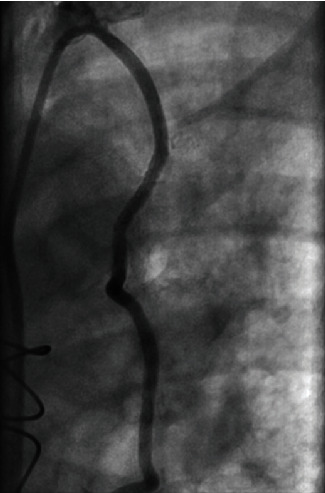
Postintervention angiography showing no residual aneurysm or stenosis in the left internal mammary artery bypass graft.

As for the large aneurysm found in the LIMA, it was decided that it would either be treated by surgical repair or by percutaneous intervention at a later date. The patient was discharged home to continue dual antiplatelet therapy and start aggressive blood pressure and lipid control. Three weeks later, the patient returned to the hospital to undergo repair of the large aneurysm found in the LIMA to LAD bypass graft. Following multiple discussions with and evaluation by cardiothoracic surgery, it was concluded that the patient would best benefit from placing a covered stent to repair the aneurysm. Intravascular ultrasound was used to determine the size and length of the lesion. A 2.8 × 19 mm covered stent was placed in the midsegment of the aneurysm (Figure 2). Postintervention angiography showed TIMI-3 blood flow with no residual aneurysm or stenosis (Figures 3 and 4). The patient was discharged the following day and instructed to continue dual antiplatelet therapy and aggressive blood pressure and lipid control.

One year later, the patient presented to the hospital with complaints of chest pain and was diagnosed with non-ST elevation myocardial infarction. During cardiac catheterization, he was found to have approximately 75% in-stent restenosis of the LIMA covered stent that was located in the midsegment of the graft. There continued to be complete resolution of the prior aneurysm. Repair within the stenosed section was performed with a 3.0 × 20 mm drug-eluting stent. Postintervention showed no residual stenosis and TIMI-3 blood flow. Ultimately, the patient was discharged home to continue life-long dual antiplatelet therapy with aggressive blood pressure and lipid control.

## 3. Discussion

Arterial aneurysms usually occur in elderly men, as well as in individuals that have uncontrolled high blood pressure [6]. They are also typically asymptomatic, small, and do not pose a significant health risk [6]. The most common clinical presentation of a symptomatic graft aneurysm is chest pain and hemoptysis [7].

### 3.1. Epidemiology

A modest number of reported cases of these aneurysms have been documented since 1978, with 51.9 years noted as a mean age of occurrence (Table 1) [8]. Aneurysms and pseudoaneurysms found in the IMA are seldomly seen outside of ailments involving hereditary diseases, infections, iatrogenic causes, vasculitis, persistent atherosclerosis, or physical trauma [9]. It is an increased likelihood that graft endothelial dysfunction, atherosclerotic changes, and changes in the orientation of the medial smooth muscle have some role in late aneurysm development [9].

**Figure 5:**
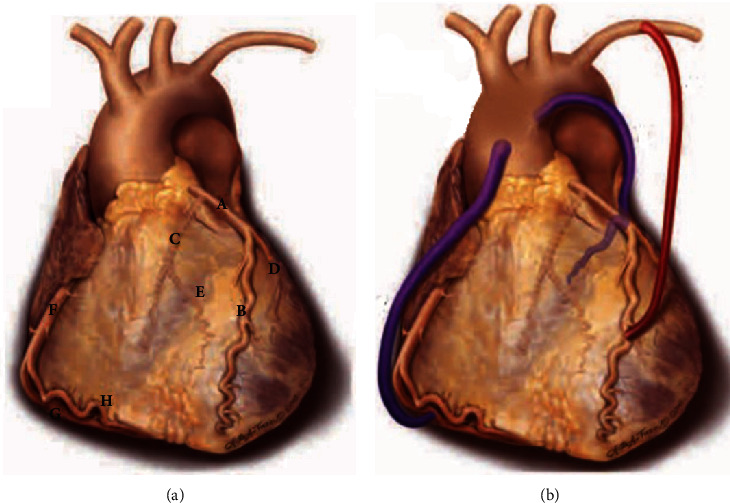
(a) Normal anatomy of the coronary arteries: (A) left coronary artery, (B) left anterior descending artery, (C) left circumflex artery, (D) diagonal artery, (E) obtuse marginal branch of the left circumflex artery, (F) right coronary artery, (G) posterior descending artery, and (H) acute marginal branch of the right coronary artery. (b) Left internal mammary artery graft from its origin at the subclavian artery proximally and grafted to the left anterior descending artery distally (in red) [4]. This figure is reproduced from Frazier, A., Qureshi, F., Read, K. M., Gilkeson, R. C., Poston, R. S., & White, C. S. (2005). Coronary Artery Bypass Grafts: Assessment with Multidetector CT in the Early and Late Postoperative Settings. Radiographics, 25(4), 881-896 (under the Creative Commons Attribution License/public domain).

IMAs are fundamentally ideal for use during bypass grafting due to a decreased tendency for spasms and reduced development of atherosclerosis [4]. Procedurally, the LIMA is removed from the chest wall and the proximal end remains attached to the subclavian artery, with the distal end newly connected to the LAD distal to the site of occlusion (Figure 5) [4]. One theory suggests that skeletonization of the IMA to preserve sternal blood perfusion in fact deprives the grafted artery of possible long-term sustainability because it adversely affects its resistance to atherosclerosis by affecting its original nerve supply, vasa vasorum, and lymphatic drainage [10]. With this theory, it may be a disadvantage to perform grafting with this method in comparison to using pedicled grafting or covered stents in a person with a known history of severe atherosclerosis [10].

### 3.2. Causes

The IMA's pregrafting location adjacent to the sternum provides increased susceptibility to penetrating injury, blunt chest trauma, and iatrogenic injury [6]. A few case reports have described the existence of these aneurysms following pacemaker lead placement or a complicated central venous catheter placement [11]. An idiopathic IMA aneurysm without a recent medical history to explain its occurrence is extremely rare [11]. IMA aneurysms can be classified into various groups such as early versus late presentation [12].

Early IMA graft aneurysms may be due to tension at the site of anastomosis or may be related to an implanted graft infection. Other causes include technical errors during anastomosis, intimal hyperplasia, direct trauma from a median sternotomy, or from atheroma formation [11]. It is likely that the patient in this case developed a LIMA aneurysm in a short time following his CABG due to iatrogenic trauma related to grafting. Late graft aneurysms are likely secondary to progressive or accelerated atherosclerotic degeneration that is due to patient medication noncompliance or resistance to treatment. IMA grafts, however, are usually resistant to the formation of atherosclerosis, which contributes to longer patency rates when compared to subclavian vein grafts [4].

### 3.3. Diagnosis

Aneurysms that are symptomatic pose a challenge diagnostically because they may present as congestive heart failure or acute coronary syndrome [12]. Having an asymptomatic clinical course, no current guidelines for screening, initial presentation of sudden death from rupture, and incidental finding on diagnostic imaging are some reasons that may contribute to the underdiagnosing of this phenomena [2]. Late presentation is also another reason that these aneurysms are likely to be missed. About 90% of saphenous vein graph aneurysms manifest greater than five years following bypass surgery, with a mean time of about 13 years before final diagnosis [2].

Reportedly, about 50% of aneurysm cases can be observed incidentally on chest X-ray and will show a hilar or mediastinal mass [4]. In one report, a pulmonologist documented that a patient that was referred to him for an evaluation of a coin lesion noted on imaging in fact was discovered to have an IMA aneurysm [5]. Other diagnostic modalities include echocardiogram, contrast-enhanced thoracic CT scan, CT angiography, and MRI angiography. The definitive imaging test for confirming an aneurysm is coronary artery CT angiography, as it will show the extent of the aneurysm, local mass effect, and will note a change in the vasculature [7]. Findings may be obscured if there is a mural thrombus involved [13]. In coming years, increased early identification of bypass aneurysms will likely be reported due to continuous improvement of long-term outcomes following coronary artery bypass grafting and due to the increasing sophistication of diagnostic investigations [6].

### 3.4. Treatment

**Figure 6:**
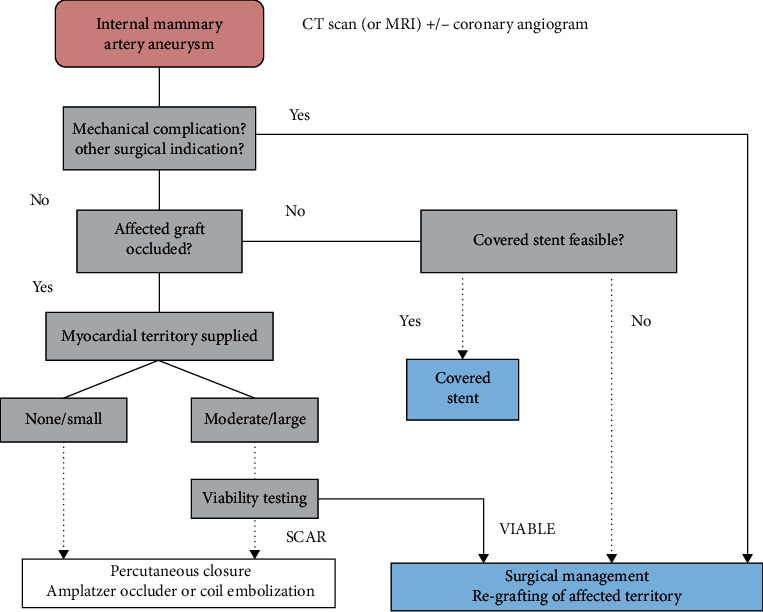
Suggested algorithm for assessment and treatment of an internal mammary artery aneurysm. CT: computed tomography; MRI: magnetic resonance imaging [7].

When a patient is diagnosed with an IMA aneurysm, it is highly recommended to begin prompt therapy to prevent life-threatening complications [11]. About 20% of patients with saphenous vein aneurysms are treated with conservative management comprising of risk factor modification, secondary prevention, and surveillance [2]. Reducing the risk of mass effect, thromboembolism, rupture, and myocardial ischemia are the main goals of treatment [2]. Options for invasive treatment are surgical repair or percutaneous approaches, which include vascular coil embolization, covered stenting, and vascular occlusion (Figure 6) [7]. Covered stenting has a major advantage in that it protects distal flow patency, unlike other percutaneous options. Surgery should be immediately considered if the patient has a high risk of rupture or if the aneurysm is causing compressive complications [2]. Surgical techniques include ligation of the aneurysm or resection of the aneurysm with or without bypass revascularization [2]. At a minimum, patients should receive a complete cardiac evaluation by an interventional cardiologist and a cardiac surgeon [7].

## 4. Conclusion

IMA aneurysms represent a very rare complication following a CABG. It has a high potential for morbidity and mortality, and the risk of complications will likely rise with the size of the aneurysm [2]. Increased attention to and awareness of this disease process will hopefully facilitate earlier diagnosis. Coronary CT angiography is the most definitive test for not only confirming but also discovering possible complications stemming from the aneurysm [13]. With continued improvement in long-term outcomes following bypass grafting since the 1960s, and with an increase in the aging population undergoing bypass surgery, it is likely that the incidence of documented IMA aneurysms or pseudoaneurysms will grow [7, 12]. Endovascular treatment is especially effective for the severely ill and elderly population, with the number of successful aneurysm cases treated with this method continuing to rise [6]. Following an extensive literature review, and to the best of our knowledge, this is the first documented case of a true aneurysm found within a LIMA bypass graft. It is our hope that this review will further assist clinicians in increasing accurate evaluation and management of this rare disease process.

## Figures and Tables

**Table 1:** Previously reported artery aneurysms [8].

Study (year)	Location	Etiology	Management	Discovery
Otter and Stam [5] (1978)	Left internal mammary artery	Unknown	Left lateral thoracotomy and ligation	“Coin lesion” on chest X-ray
Chan and Fermanis [1] (1995)	Left internal mammary artery	Unknown	Angiographic embolization	Spontaneous hemothorax
Kugai and Chibana [8] (1999)	Left internal thoracic artery	Atherosclerosis	Internal thoracic artery aneurysmectomy and reconstruction	“Angina”
Lindblom et al. [14] (2013)	Left internal thoracic artery	Unknown	Endovascular coiling	Left-sided shoulder pain radiating to chest
Heyn et al. [15] (2014)	Left internal mammary artery	Thrombotic obliterated aneurysm	Open surgical resection	Preoperative chest X-ray
Present case (2018)	Left internal mammary artery	Iatrogenic trauma during bypass grafting	Covered stent	Incidental finding during cardiac catheterization

## Data Availability

Data is available upon request to the corresponding author.
